# Multimorbidity and Comorbidity of Chronic Diseases among the Senior Australians: Prevalence and Patterns

**DOI:** 10.1371/journal.pone.0083783

**Published:** 2014-01-08

**Authors:** M. Mofizul Islam, Jose M. Valderas, Laurann Yen, Paresh Dawda, Tanisha Jowsey, Ian S. McRae

**Affiliations:** 1 Australian Primary Health Care Research Institute, Australian National University, Canberra, Australian Capital Territory, Australia; 2 Health Services and Policy Research, University of Exeter Medical School, Exeter, England, United Kingdom; Federal University of Rio de Janeiro, Brazil

## Abstract

Understanding patterns and identifying common clusters of chronic diseases may help policymakers, researchers, and clinicians to understand the needs of the care process better and potentially save both provider and patient time and cost. However, only limited research has been conducted in this area, and ambiguity remains as those limited previous studies used different approaches to identify common clusters and findings may vary with approaches. This study estimates the prevalence of common chronic diseases and examines co-occurrence of diseases using four approaches: (i) identification of the most occurring pairs and triplets of comorbid diseases; performing (ii) cluster analysis of diseases, (iii) principal component analysis, and (iv) latent class analysis. Data were collected using a questionnaire mailed to a cross-sectional sample of senior Australians, with 4574 responses. Eighty-two percent of respondents reported having at least one chronic disease and over 52% reported having at least two chronic diseases. Respondents suffering from any chronic diseases had an average of 2.4 comorbid diseases. Three defined groups of chronic diseases were identified: (i) asthma, bronchitis, arthritis, osteoporosis and depression; (ii) high blood pressure and diabetes; and (iii) cancer, with *heart disease and stroke* either making a separate group or “attaching” themselves to different groups in different analyses. The groups were largely consistent across the approaches. Stability and sensitivity analyses also supported the consistency of the groups. The consistency of the findings suggests there is co-occurrence of diseases beyond chance, and patterns of co-occurrence are important for clinicians, patients, policymakers and researchers. Further studies are needed to provide a strong evidence base to identify comorbid groups which would benefit from appropriate guidelines for the care and management of patients with particular disease clusters.

## Introduction

Multimorbid and comorbid chronic diseases are increasingly placing a greater burden on individuals, communities and health care services. With ageing of the population and longer survival, scientific advances in medical care and public health policy, a growing proportion of the population is surviving longer with multiple chronic diseases. It is also increasingly recognised that diseases tend to occur together leading to a rising interest in the ‘common pathways’ implicated in the clustering of diseases and required responses to the potential to help better organise medical responses [1]–[3]. This co-occurrence of diseases has implications from a disease management point of view, as the features of comorbid diseases can be much more complicated than a simple aggregation of individual illnesses [4]. It also has implications for studies which explore the implications of chronic diseases, as for many people the impact of multiple diseases compound and interact. Hence using a single-morbidity model – where the impact of a single disease is explored – may mean one fails to grasp the pattern of disease, leading to inadequate clinical management, or to inadequate understanding of the disease effect by researchers and policymakers.

Although the need to understand the patterns of disease combinations/clusters and associated complexity and care is well recognised [5], research conducted on these issues remains limited. Concern exists about increased time and cost requirements for both the individual and the health care system [6]–[8] caused by comorbidities. Identifying common clusters may improve understanding of these effects and enable policymakers and clinicians to work towards simplifying the care process, and saving patients time and costs. A recent study of working Australians found multimorbidity is increasingly prevalent in Australia [9]. A systematic review of Australian studies on multimorbidity endeavoured to identify prevalent groups of co-occurring diseases and found almost a third of the studies included scored only 50% using the critical appraisal tool, highlighting the need for increased research with greater methodological rigour [10].

Studies of comorbidity have used different analytic approaches, some used simple disease counts, some performed cluster analysis and others used factor analysis or correspondence analysis [11]–[16]. Use of different approaches has led to a fragmented and incomplete understanding of the nature and impact of multimorbidity. Moreover, study findings may vary with approaches [17]. Problems also arise in comparing methods across studies as some are based on self-report and some on clinical records, some use very comprehensive lists of diseases and some relatively few diseases. Few of those studies increased the reliability of their findings by comparing methods to establish difference in outcomes using different approaches [18]. Existing literature reveals no consensus about how the co-occurrence of diseases should be measured [19], [20]. There remains a clear lack of an internationally accepted standard for assessing which diseases are likely to co-occur.

This study aimed to identify the pattern of co-occurrence of common chronic diseases in senior Australians, and also to explore whether and how the patterns identified differed if different analytic methods were used. By applying different methods to a single data set, issues of data collection and comprehensiveness of lists of conditions are avoided. A high degree of consistency in the results would provide confidence in the patterns, a lack of consistency would lead to questions about the “best” approach.

This study used four analytical approaches: (i) identifying the most frequently occurring pairs and triplets of comorbid diseases; performing (ii) cluster analysis of diseases, (iii) principal component analysis, and (iv) latent class analysis. The paper also examines the possible methodological reasons for consistency (or variation) in findings across the methods. We go on to discuss comorbid patterns from the point of clinical epidemiology, and question the way in which this knowledge might inform clinical management.

## Methods

### Setting and respondents

The study population are members of the National Seniors Australia, a nation-wide organisation with 285,000 members aged 50 years and over. An opt-in invitation and a study questionnaire were mailed to a representative cross-section of their membership base (*n* = 10,000) during mid-2009. The questionnaire was piloted and revised before mailing to the respondents. The sample was stratified by age, rurality and state of residence, with those aged 75 years or older over-represented to permit analysis of this older cohort. Survey questions were drawn from existing validated tools (for details see McRae et al., [6]). The survey and study were approved by the Australian National University Human Research Ethics Committee (no. 2009/309). All respondents provided informed consent to participate by returning completed questionnaires. The Ethics Committee approved this consent procedure.

Data about chronic illnesses were collected using a list of 11 diseases (Table 1). Respondents were asked ‘Has a doctor ever told you that you had any of the following illnesses?’ This was followed by the list of diseases. The final open question asked the respondent to nominate any other long-term condition that had been diagnosed. This study focused on a sub-set of those conditions comprising the most common serious chronic diseases in Australia [21]: cancer, heart disease, high blood pressure (HBP), stroke, asthma/hayfever, bronchitis/emphysema, diabetes, arthritis, osteoporosis, Parkinson's disease, depression (including anxiety).

**Table 1:** Prevalence of Selected Chronic Diseases.

Diseases	n	Weighted prevalence (%)	% with comorbidity	Co-occurring diseases mean±SE^A^
Cancer	868	17.9	84.8	2.1±0.05
Heart disease	724	12.3	90.1	2.3±0.06
High blood pressure	2047	43.1	82.1	1.8±0.03
Stroke	179	3.2	96.1	2.8±0.12
Diabetes	563	12.8	89.7	2.2±0.06
Asthma/hayfever	773	18.2	88.0	2.2±0.05
Bronchitis/Emphysema	191	3.4	97.4	3.0±0.11
Arthritis	1597	32.2	87.7	2.0±0.03
Osteoporosis	531	9.3	90.8	2.3±0.06
Parkinson's disease	39	0.60	89.7	2.6±0.27
Depression and anxiety	625	15.3	92.8	2.4±0.06
Other	1218	25.4	87.8	2.0±0.04

^A^ other comorbid diseases apart from those mentioned in column 1.

All prevalence estimates are weighted to reflect the age, sex, and State structure of the Australian population. We used four approaches (expected/observed prevalence ratios, cluster analysis, principal component analysis and latent class analysis) to explore whether diseases were independent of each other, and if not, whether there are any common patterns of grouping across the approaches. We did not use any weighting in these analyses.

### Prevalence of comorbidity and probability of particular groupings

The presence of disease is reported as frequency and prevalence (per 100 persons) of occurring (independent of any comorbidity). The mean number and standard error of co-occurring diseases (apart from the index condition) were estimated.

The prevalence of the most common disease pairs with observed prevalence of ≥5% was estimated, and whether these prevalences were greater than would be expected if the two diseases were independent was tested using a Chi-square test, and further tested by logistic regressions between each pair of co-occurring diseases with and without adjustment for age, sex, education, and all of the other diseases. Ratios between *observed* versus *expected* were also calculated and are reported. Similarly a list of three diseases (triplets) that coexisted within the same respondent was examined and most common triplets were counted in terms of absolute frequency and per 100 respondents. Chi-square testing was undertaken with the triplets to assess whether observed prevalences differ from expected ones, but no logistic regression was conducted.

### Method of clustering

Data on chronic diseases were collected as binary objects, taking the value of ‘1’ when a given disease was present and ‘0’ when it was absent. Our basic interest is in identifying clinically meaningful clusters of chronic illnesses based on their relative similarity or dissimilarity (also known as distance). Our dataset is a collection of binary objects arranged in an n×p matrix with rows representing the n ( = 4574) respondents and columns representing the p ( = 11) chronic diseases. The classical approach to cluster analysis is to classify n respondents into a set of clusters based on index of proximity among the respondents, yielding an n×n proximity matrix reflecting the degree of closeness among the respondents to see if they comprise clusters of diseases. However, it is also possible to cluster variables (chronic diseases) and produce groupings of chronic diseases based on the relative proximity of variables. The problem simplifies to reducing the transposed p×n data matrix to a much smaller p×p proximity matrix among the chronic illness diseases, rather than a potentially large n×n proximity matrix [11]. We have undertaken both clustering approaches.

As many chronic diseases share the same underlying genetic, environmental or behavioural risk factors, analysing clusters of variables using the hierarchical clustering approach was appropriate. Under this method, each individual disease begins as an individual cluster which is gradually merged with the most closely related other clusters until a single cluster containing all comorbidities is obtained. We chose this agglomerative approach as we did not know the possible number of clusters a priori, and the number of clusters was assessed using a dendrogram, and agglomerative coefficient. To measure the distance between two clusters we used the average linkage method [22] to accommodate the spread of the clusters.

In the context of cluster analysis for grouping of observations a partitional clustering with k-medoids was performed. This clustering process starts by randomly assigning objects to a number of clusters. Unlike the hierarchical clustering approach, where an object remains in a cluster once it is assigned to it, the k-medoids proceeds with iteration. The objects are then successively reassigned to other clusters to minimize the within-cluster variation. If the reallocation of an object to another cluster decreases the within-cluster variation, this object is reassigned to that cluster, and this iteration continues until it reaches to the least within cluster variation [23]. We used STATA cluster stopping rule with the Calinski–Harabasz pseudo-F index to determine the appropriate number of groups [24].

For cluster analysis (both for observation and variable clustering) with binary data a number of similarity measures have been used in the literature; Jaccard coefficient and Yule's Q were commonest among them. The choice of similarity measure depends on the relative weight given to positive and negative matches, which in turn depends on the relative importance of positive and negative matches [22]. In the Jaccard similarity, negative matches (in a 2×2 table the frequency of the cell that presents group having neither of the diseases) are virtually non-informative [11] and receive zero weight, whereas positive matches and non-match elements receive equal weights. However, negative matches are considered informative in Yule's Q. In fact, negative matches are also part of calculation of tetrachoric correlations used in our principal component analysis. As one of our aims is to see if different approaches produce same results, to ensure consistency across the approaches, we used the Yule's Q as the similarity measure in both forms of cluster analysis.

Chronic diseases with very low prevalence (<2.0%) were excluded from analyses to minimise sequential joining of low prevalence comorbidities into existing clusters [22]. As part of checking stability the dataset was split into two halves and the two subsets were analysed separately using the same parameter settings. Sensitivity of clusters/groups was also tested by observing changes (if any) of pattern of clusters due to exclusion of individual diseases from the analysis.

### Principal component analysis

A standard principal component analysis was performed with a varimax rotation applied to facilitate interpretation of component loadings. The aim of this analysis is to summarize the observed variables into a reduced set of variables. As the variables are dichotomous the analysis was based on a correlation matrix populated with tetrachoric correlations which are more appropriate than Pearson correlations in this context [25]. The optimal number of components was determined using a number of indices including the scree test, the Eigenvalues-greater-than-one rule, standardized root mean square residual; comparative fit index and Tucker Lewis index. The criterion for factor loading was set at ≥0.30.

### Latent Class Analysis

Latent class analysis was used to classify objects or individuals according to their distribution on 10 chronic diseases. Like cluster analysis, it is aimed at identifying clusters (classes) of individuals that are in some sense ‘similar’. However, there is no need to define cluster distance (or similarity), nor to select cluster algorithms (e.g. agglomerative); rather latent class analysis classifies objects according to the probabilities of the observed values of all variables for each object [26]. For identifying an optimal baseline model a sequence of models was examined with two classes, three classes, and so on. A range of indices was used for model selection, including the likelihood-ratio *G* ^2^ statistic, and Bayesian Information Criterion (BIC). In addition, model interpretability was considered, for example, distinguishability of each class from the others on the basis of the item-response probabilities, triviality in size (i.e., no class should have a near-zero probability of membership), and the possibility of assigning a meaningful label to each class.

An iterative maximum likelihood estimate was used, which requires ‘random’ starting values. The estimate was repeated with a different set of ‘random’ starting values. Models were identified that had a frequently occurring dominant solution. Solutions were considered to be identical if the log likelihood and parameter estimates were replicated [26].

Data were analysed using STATA (version 12), SPSS (version 20) and SAS (9.3).

## Results

### Demographic characteristics

A total of 4,574 people returned the completed survey. The overall response rate was 45.7%, with little difference between male and female response rates (45.1% to 46.3%). The average age of respondents was 69.3 years. Only 15 respondents identified themselves as of Aboriginal or Torres Strait Islander descent. Most respondents (77%) were born in Australia. More than half of the respondents had post-school qualifications. Sixty percent were completely retired or pensioners. Eighty percent had current private health insurance. The study sample was similar to the Australian population on most of the demographic characteristics except that the sample members were better educated, reported better health and were more likely to have private insurance coverage than the average Australian in their age range. The estimated prevalence of chronic disease in the study population was also similar to the Australian population prevalence in this age group, although respondents reported a higher prevalence of high blood pressure, history of cancer diagnosis and a lower prevalence of arthritis [6].

### Prevalence of chronic diseases and comorbid diseases

Eighty-two percent of respondents reported having at least one chronic disease and over 52% having at least two chronic diseases. Female respondents reported a significantly higher number of diseases than male respondents. Of those respondents aged over 75 years, 93% experienced at least one chronic disease and 73% more than one chronic disease. Overall, 27% reported at least three chronic diseases, 11% at least four and 3% at least five diseases. High blood pressure (HBP) (43.1%), arthritis (32.2%) and cancer (17.9%) were three most prevalent diseases (Table 1).

Respondents who had any chronic disease had an average of 1.5 (SE±0.02) additional comorbid diseases. The number of comorbid diseases varied from an average of 1.8 to 2.6 between the various index conditions (last column, Table 1). Comorbidity was highest among those with chronic bronchitis/emphysema (97%) and lowest among those with HBP (82%).

Table 2 presents the observed and expected prevalence of the most frequently co-occurring pairs of diseases and their crude and adjusted odds ratios. Most diseases of these pairs were identified as the most prevalent in Table 1. Six of the eleven pairs with a prevalence of over 5% show a statistically significant relationship (meaning more are observed than would be expected at random from the prevalence of the components of the pairs). *HBP and diabetes* were the pair of diseases with the strongest association reflected by the adjusted odds ratio followed by *arthritis and depression*, and *asthma/hayfever and arthritis*. Adjustment for age, gender, education, income, and region did not influence the relationships with the larger odds ratios but did change a number of weaker effects (in both directions).

**Table 2:** Persons affected by the most frequently co-occurring pairs of chronic diseases and their observed and expected prevalence per 100 population.

Frequent co-occurring pairs	n	Prevalence/100			Chi^2^ (*p* value)^B^	Odds Ratio (from Logistic regression) (95% CI)	
		Observed	Expected^A^	Observed/Expected		Crude	Adjusted
HBP and Arthritis	826	18.05	15.62	**1.16**	**9.8 (<0.01)**	**1.5 (1.4–1.7)**	**1.4 (1.2–1.6)**
HBP and Cancer	401	8.77	8.49	1.03	0.2 (0.63)	1.1 (0.9–1.2)	0.9 (0.8–1.1)
HBP and Heart disease	381	8.33	7.08	**1.18**	**5.0 (0.02)**	**1.5 (1.2–1.7)**	1.1 (0.9–1.3)
Asthma/hayfever and arthritis	367	8.02	5.90	**1.36**	**15.9 (<0.01)**	**1.9 (1.6–2.2)**	**1.6 (1.4–1.9)**
HBP and Diabetes	365	7.98	5.51	**1.45**	**22.2 (<0.01)**	**2.5 (2.1–3.1)**	**2.4 (2.0–2.9)**
HBP and Asthma/hayfever	348	7.61	7.56	1.01	0.01 (0.94)	1.0 (0.9–1.2)	1.0 (0.8–1.2)
Cancer and Arthritis	319	6.97	6.62	1.05	0.44 (0.51)	1.1 (0.9–1.3)	1.0 (0.8–1.2)
Arthritis and Depression^C^	307	6.71	4.77	**1.41**	**16.0 (<0.01)**	**2.0 (1.7–2.4)**	**1.9 (1.6–2.3)**
HBP and Depression^C^	304	6.65	6.11	1.09	1.1 (0.28)	1.2 (1.0–1.4)	**1.2 (1.0–1.5)**
Heart disease and Arthritis	284	6.21	5.51	1.13	2.0 (0.15)	**1.2 (1.1–1.5)**	1.1 (0.9–1.3)
Arthritis and Osteoporosis	257	5.62	4.05	**1.39**	**12.3 (<0.01)**	**1.9 (1.6–2.3)**	**1.3 (1.1–1.6)**

^A^ Multiplication of observed prevalences of individual disease;

^B^ Test of independence for observed and expected prevalences;

^C^ including anxiety.

### Leading multimorbid triplets

The weighted prevalence of three way combinations of diseases (triplets) shows that the three most common triplets are *HBP, asthma/hayfever and arthritis* (4.3%), *HBP, arthritis and depression* (3.7%) and *cancer, HBP and arthritis* (3.5%) (Table 3). Clearly, the most common triplets were determined by respondents having highly prevalent chronic diseases such as HBP and arthritis (all 15 triplets had either of these two diseases and five triplets have both of them). The ratio of observed to expected value was highest for the *asthma-arthritis-depression* triplet, followed by the *heart disease-HBP-diabetes* (Table 3).

**Table 3:** Most prevalent triplets (three ways disease combinations).

Order	Cancer	Heart	HBP	Stroke	Diabetes	Asthma/Hayfever	Bronchitis/Emphysema	Arthritis	Osteoporosis	Depression (& anxiety)	Combination, n (%)	Prevalence	Observed÷Expected	Chi^2^ (*p* value)^A^
1			X			X		X			192 (4.2)	4.3	1.6	16.7 (<0.01)
2	X		X					X			179 (3.9)	3.5	1.3	6.1 (0.01)
3		X	X					X			175 (3.8)	3.2	1.5	13.8 (<0.01)
4			X					X		X	164 (3.6)	3.7	1.7	17.1 (<0.01)
5			X		X			X			155 (3.4)	2.8	1.8	19.0 (<0.01)
6						X		X		X	105 (2.3)	2.7	2.8	33.1 (<0.01)
7	X	X	X								102 (2.2)	1.9	1.7	15.5 (<0.01)
8		X	X		X						88 (1.9)	1.5	2.2	18.3 (<0.01)
9			X			X				X	88 (1.9)	2.3	1.9	12.6 (<0.01)
10	X	X						X			79 (1.7)	1.7	1.6	7.7 (<0.01)
11	X		X			X					79 (1.7)	1.8	1.2	1.2 (0.28)
12	X		X		X						74 (1.6)	1.3	1.5	5.6 (0.02)
13	X					X		X			73 (1.6)	1.8	1.4	4.0 (0.05)
14	X							X		X	71 (1.6)	1.6	1.5	8.1 (<0.01)
15		X	X			X					69 (1.5)	1.4	1.2	1.6 (0.21)

^A^ Test of independence for observed and expected prevalences.

### Cluster analysis

#### (i) Variable clustering approach

**Figure 1:**
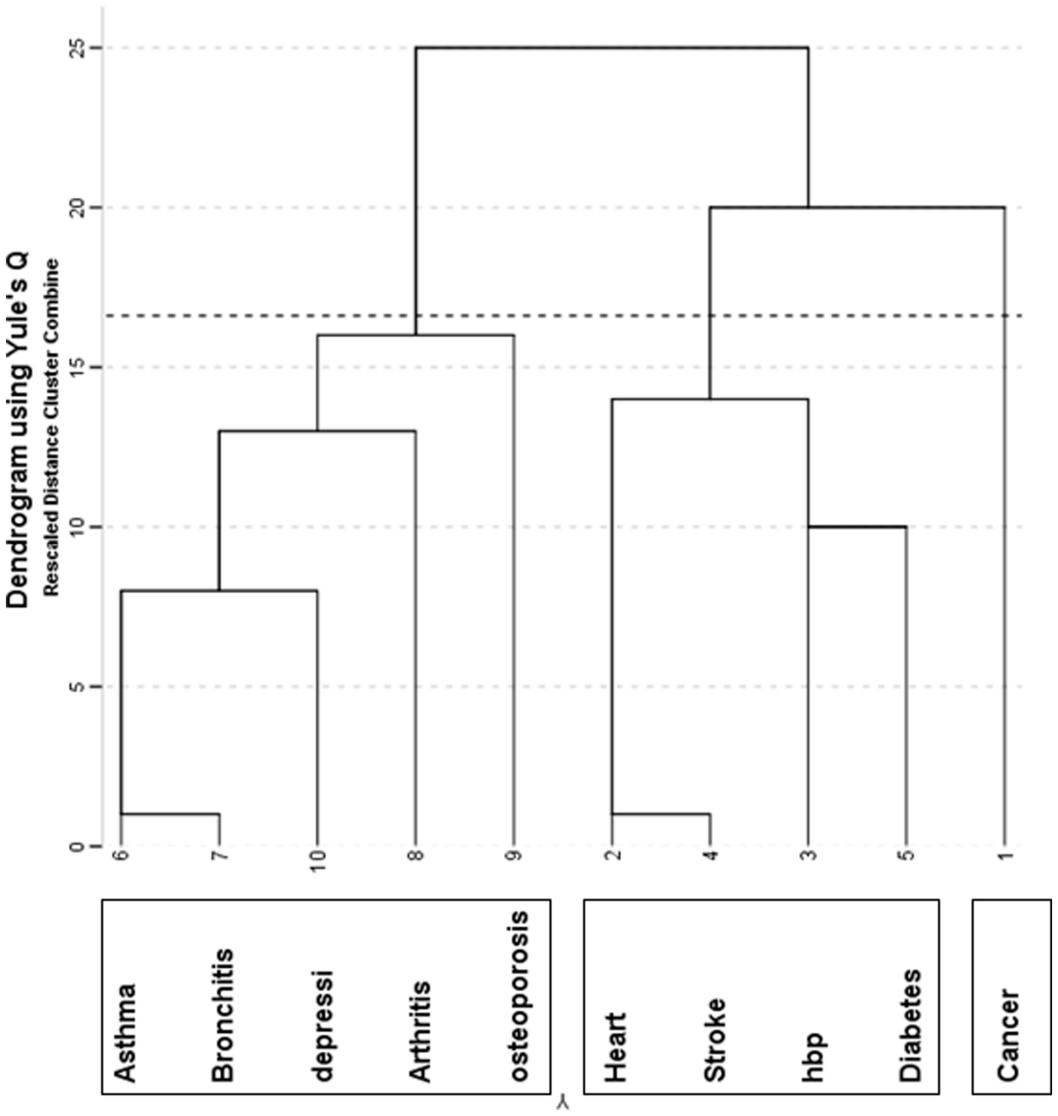
Dendrogram of likely clusters using variable clustering of 10 chronic diseases.

As the prevalence of Parkinson's diseases was less than 2%, it was not included in any further analysis. Figure 1 presents a dendrogram of the variable based cluster analysis using average linkage and Yule's Q similarity measure. Stepwise agglomerative coefficients suggest a three cluster solution is most feasible. The change in the agglomerative coefficient when stepping from 3 to 2 groups is at least twice as large as for any other step. *Heart disease* and *stroke* had the smallest distance and thus formed the first cluster, which joined to another cluster comprised of *HBP and diabetes*, and finally are reflected as a four-disease cluster (*heart disease, stroke, HBP and diabetes*). *Asthma and bronchitis* formed the second cluster which then joined by *depression* at a relatively higher distance. *Arthritis and osteoporosis* then added to that cluster in the next step, finally making a five-disease cluster. Cancer alone runs all the way through the process without linking with other diseases until it merges to the *heart disease-stroke-HBP-diabetes* cluster at a relatively large distance, meaning cancer becomes part of this cluster at a very low similarity value. The three cluster solution suggested by dendrogram would be:

- Cluster 1: asthma, bronchitis, arthritis, osteoporosis and depression (including anxiety)
- Cluster 2: heart disease, stroke, HBP and diabetes
- Cluster 3: cancer

#### (ii) Cluster of observations

Following the partitional k-medoids approach four groups were identified (Table 4). These groups were identified by combinations of diseases, and we now label the clusters which we call Groups to distinguish from variable cluster according to the dominant diseases in each group. For instance, 46% of the respondents with HBP fell in Group 2 with the rest in Group 1 (23%), Group 3 (16%) and Group 4 (15%). Thus for the purpose of clustering respondents with HBP were labeled as belonging to Group 2.

**Table 4:** Identification of groups in k-medoids clustering approach.

Disease	n	% of respondents belong to individual groups			
		Group1	Group2	Group3	Group4
Cancer	868	0	1	18	**82**
Heart	724	3	3	**91**	3
HBP	2047	23	**46**	16	15
Stroke	179	19	25	**41**	15
Diabetes	563	9	**59**	19	13
Asthma	773	**38**	37	**7**	17
Bronchitis	191	**36**	28	19	17
Arthritis	1597	**64**	5	15	16
Osteoporosis	531	**53**	16	14	18
Depression and anxiety	625	**51**	21	11	17
**% total in each group**		**26.4**	**43.4**	**14.7**	**15.5**

Group 2 was also the dominant group for diabetes, and hence we describe Group 2 as *HBP and diabetes*. Similarly, for asthma, bronchitis, arthritis, osteoporosis and depression the dominant group was Group 1, while heart disease and stroke identified Group 3 and cancer alone formed a separate group. Percentages of observation in each of the four groups are shown in Table 4.

### Principal component analysis

**Figure 2:**
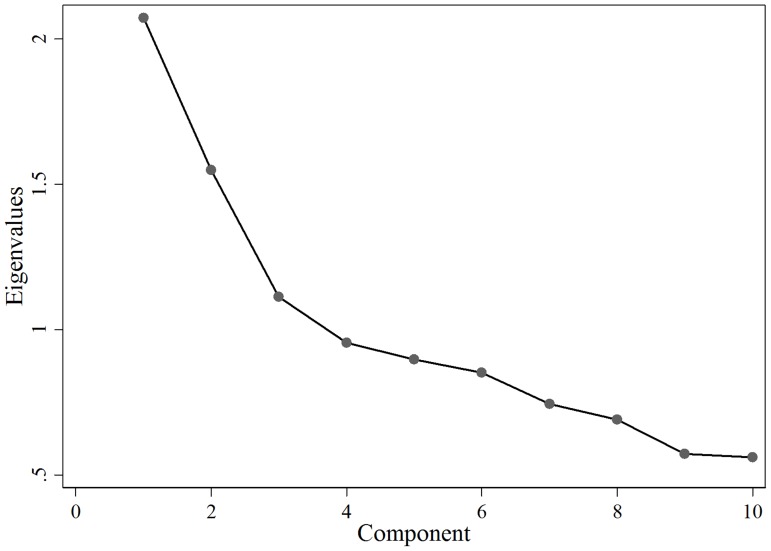
Scree test with Eigenvalues for range of factors.

Iterations produced three Eigenvalues greater than 1 as shown in the scree chart (Figure 2). All of other indices also suggest a three components solution. Loadings exceeding the cut-off ±0.30 are reflected in Table 5. The following three components were identified with loading >±0.30:

**Table 5:** Loadings with values >|0.3|.

Variable	Component 1	Component 2	Component 3
Cancer		0.4740	
Heart		0.6091	
HBP			0.6041
Stroke		0.5136	
Diabetes			0.6288
Asthma	0.5462		
Bronchitis	0.4536		
Arthritis	0.4282		
Osteoporosis	0.3063		
Depression and anxiety	0.4497

- Component 1: asthma, bronchitis, arthritis, osteoporosis and depression (including anxiety)
- Component 2: cancer, heart disease and stroke
- Component 3: HBP and diabetes

The three principal components we identified do not have any overlapping diseases and give us three clear clusters of diseases. Of the 10 diseases available for analysis in our study, we found loadings were highest for *HBP and diabetes* followed by those for *heart disease and stroke*.

### Classes identified using latent class analysis

The drop in *likelihood ratio G* ^2^ relative to the drop in degrees of freedom is substantial with each additional class up to the four-class model; the addition of classes beyond four provides essentially no improvement in fit. Adjusted BIC values (one-class model: 1361; two-class: 1025; three-class: 916; four-class: 909; five-class: 932; six-class: 961) agreed with the G^2^ statistics. Thus four latent classes were identified, and labelled as (i) relatively healthier group, (ii) group with dominant presence of arthritis, asthma and depression, (ii) group with dominant presence of HBP and diabetes, and (iv) group with dominant presence of cancer, heart and stroke. Very small values of item-response probabilities (rho parameters) of ‘relatively healthier’ group suggest none of the 10 diseases are prevalent in this group. However, relatively higher values of item-response probabilities for other three groups suggest these diseases are strongly associated with each other in those three individual groups. Item response probabilities of bronchitis and osteoporosis are same in two groups (column 3 and 5, Table 6). Based on the clinical nature of the diseases, we have grouped bronchitis with asthma and osteoporosis with arthritis. Thus, in terms of grouping of diseases we found three meaningful classes, as described above.

**Table 6:** Item-response probabilities for four class model: probability of individual diseases in latent class.

Item	Relatively healthier	Sick group with dominant presence of arthritis, asthma and depression	Sick group with dominant presence of HBP and diabetes	Sickest group with dominant presence of cancer, heart and stroke
% of respondents in the group	55.5	19.4	13.0	12.1
Cancer	0.17	0.17	0.13	**0.34**
Heart	0.12	0.04	0.13	**0.54**
HBP	0.27	0.42	**0.99**	0.69
Stroke	0.01	0.01	0.05	**0.19**
Diabetes	0.07	0.07	**0.32**	0.23
Asthma	0.08	**0.44**	0.06	0.23
Bronchitis	0.00	**0.11**	0.01	0.12
Arthritis	0.21	**0.60**	0.38	0.55
Osteoporosis	0.07	**0.23**	0.04	0.23
Depression and anxiety	0.06	**0.31**	0.13	0.20

Table 7 presents probable clusters identified through two cluster analysis methods, principal components, latent class analysis and the top three associated triplets. The patterns of the clusters which emerged from the two clustering methods appear to be similar in terms of disease grouping, except for *heart disease* and *stroke* which, while always together, form a separate group in observation based clustering, and change group in principal component analysis. Notably, in both clustering methods cancer sits alone separately, but is grouped with *heart disease and stroke* in the principal component analysis. There is a great similarity between the groups found from clustering of observations and principal component analysis, again the exception being *heart disease and stroke*. There was also consistency between the groups identified using principal component analysis and latent class analysis. Overall, Table 7 suggests there are three well defined groups of chronic diseases: (i) *asthma, bronchitis, arthritis, osteoporosis and depression*; (ii) *HBP and diabetes*, and (iii) *cancer*, with *heart disease and stroke* either making a separate group or ‘attaching’ themselves to different groups in different analyses.

**Table 7:** Highly associated triplets, likely clusters, three principal components and classes identified using latent class analysis.

Three highly associated triplets			Clustering of variables			Clustering of observations				Three principal components			Groups identified using latent class analysis		
			Three clusters using Yule's Q similarity measure			Three clusters using k-medoids and Yule's Q similarity measure									
AsthmaArthritisDepression	HBPHeartDiabetes	HBPAsthmaDepression	AsthmaBronchitisArthritisOsteoporosisDepression	HeartStrokeHBPDiabetes	Cancer	AsthmaBronchitisArthritisOsteoporosisDepression	HBPDiabetes	HeartStroke	Cancer	AsthmaBronchitisArthritisOsteoporosisDepression	CancerHeartStroke	HBPDiabetes	AsthmaBronchitisArthritisOsteoporosisDepression	CancerHeartStroke	HBPDiabetes

Depression includes anxiety.

In the sensitivity test exclusion of individual diseases from the analysis did not change the patterns of grouping in variable clustering, principal component analysis or latent class analysis. However, in clustering observations, low prevalence diseases such as stroke were found to sometimes move to another group when some diseases were omitted, and diseases for which one group was only marginally dominant over another (e.g. 38% of asthma respondents belongs to Group1 and 37% to Group2) sometimes moved to the group that had the second highest number of respondents. As mentioned earlier, for checking stability, the dataset was split into two halves; separate analyses with those two subsets using the same parameter settings produced consistent grouping of diseases.

## Discussion

This study confirms that HBP and arthritis, the two leading chronic diseases, are dominant in major comorbid pairs and multimorbid triplets among older Australians. As observed, while some pairs and triplets are more prevalent than would be the case if the diseases were independent, the measurements based on pairs and triplets are mostly guided by the prevalence of the individual diseases, and they are mainly important for identifying the most numerous groups of patients. Findings from the multivariate approaches regarding patterns of comorbidity were largely consistent, even when the dataset was split into two halves and after exclusion of individual diseases. Overall, our study demonstrates that while different analytical methods can lead to somewhat different associations; there is broad consistency in associations across the multiple modes of analysis. In general, it is difficult to compare our results with findings of other studies of similar type because there remain variations in data sources and structures, populations and diseases studied [27]. However, overall prevalence of comorbidity and multimorbidity of our study are consistent with the reported range of multimorbidity rates in elderly populations [28]–[30].

While the results provided in Table 7 show considerable consistency across the analytic methods, there are some differences which reflect the different analytic approaches. The major difference in the methods is between the cluster analyses which are based on distance measures, and the principal components and latent class analyses which are based on correlations. The results in the latter two approaches are in fact the same, but are different from the distance-based approach in that cancer is in a group of its own, while in the correlation-based groups cancer is linked with heart disease and stroke. This may arise because the proportion of participants with the *heart disease and stroke* pair who reported having cancer was 31%, which is higher than the proportion of cancer reported by the participants with other pairs.

Within the cluster analyses, since we used same distance measure (Yule's Q) for both approaches, it is not surprising that the results are quite similar, with the only difference being that the heart-stroke-HBP-diabetes group in the variable clustering approach is split in the observation clustering approach (see Table 7). Looking at the dendrogram in Figure 1 we see that this group even in the variable clustering process comprises the same two pairs of conditions as are found in the observational clustering. The different approaches therefore basically generate similar groupings, but the nature of the “cutoffs” lead to slightly different final groups in our study. Despite utilising different methods (agglomerative hierarchical clustering and k-medoids) which approach the problem from different angles, we found consistency in the groupings.

As mentioned earlier, *heart disease and stroke* formed a cluster in their own right in the hierarchical method and were found strongly correlated in the principal component analysis. The most strongly associated comorbid pair in principal component analysis was *HBP and diabetes.* This outcome is consistent with those from previous studies; that these conditions share significant underlying risk factors and associated common complications [31]. Another strongly associated pair – *arthritis and depression* – is also supported by an increasing body of research, although the causality mechanism by which arthritis may lead to depression or vice versa remains unclear [32].

Some diseases (e.g. heart disease and stroke) appear in different groupings in the different analyses, and this is clinically feasible, as there could be same underlying factors for a number of chronic diseases [1]. However, the number and the overall pattern of clusters (Table 7) are broadly consistent. In both of the clustering approaches we found cancer to stay in a separate cluster. This appears to be reasonable as cancer is a disparate group of diseases [18]. Although the most common risk factors for cancer, such as age, smoking, poor diet, obesity and physical inactivity, are shared by many other comorbidities of interest [33], risk factors for a specific cancer may be unique.

One of the major strengths of our study is that we used a range of analytical methods, and that our dataset was relatively large. Whatever consistency (or discrepancy) we observed was validated by the findings of four different approaches. In cluster analysis subject-expertise and judgment are often needed for assessing number and consistency of clusters. Our use of several statistical approaches reduced the heavy reliance on subjective judgment. Although data for the chronic diseases were binary, the use of tetrachoric correlations for principal component analysis addresses this concern. The observed degree of differences in results between the approaches is mostly explained by the underlying statistical formulae. Our use of latent class analysis reduced the reliance on choice of similarity measure in cluster analysis, the choice of which is often subjective in the literature. There were, of course, some minor judgments to be taken in the latent class analysis. The consistency of groups identified through the latent class analysis further substantiated the findings and supported the stability of the grouping of diseases.

It is difficult to directly compare our findings with those of the previous studies since the results depend on a range of factors including number and type of diseases included, the demographic and underlying risk-factors of the sample, and the mode of collection of the information. Despite this complexity, meaning that results will be different, it is important to present some of our findings in relation to both prevalence of particular comorbidities and the structure of comorbidity alongside the findings of selected previous Australian studies. In a study of the consultations provided by a sample of Australian general practitioners in 2005, Britt et al. [17] found that combination of arthritis/chronic back pain and vascular disease was the most common comorbidity (15.0% of sample). Our observation that the most common combination was HBP and arthritis (18.05 of sample) reflects broadly similar conditions. Age is likely to be one of the major factors explaining the differences between our study and that of Britt et al. [17], who studied all the patients attending a GP irrespective of their age, whereas our respondents were aged 50 years or older. In a systematic review with studies of chronic diseases among the elderly population in Australia, Caughey et al. [10] reported that over half of the elderly patients with arthritis also had hypertension and over 60% of patients with asthma reported arthritis as a comorbidity. Our observations are similar to the former combination, although a little less than the latter.

In a study of working Australians, Holden et al. [9] identified six clinically meaningful groups and found that observed clusters did not fall neatly into organ systems, and some diseases appeared in more than one cluster. There was a certain degree of similarity between the findings of Holden et al., (2001) and our study. For instance, asthma and chronic obstructive pulmonary disease were found to have been in a group, together with allergies which we did not identify as a separate disease, and like our study HBP and diabetes were found to remain together.

The difficulty in comparing findings from studies concerning comorbidity and multimorbidity is of central concern to policymakers and clinicians, who seek to improve health service delivery and management of people living with comorbid and multimorbid illness. Our study offers multiple methodological approaches to understanding the associations between specific diseases, which is a first and essential step towards enabling policymakers and clinicians to reach their goal. While our study demonstrates that different analytical methods can lead to different associations, it also demonstrates broad consistency in associations across multiple modes of analysis. Future initiatives to improve policy and service delivery for management of comorbidity and multimorbidity should therefore pay close attention to the methods employed in research that underpin policymaker and clinician decision making. If studies can demonstrate that associations remain strong using multiple modes of analysis this will strengthen the validity of study findings, and better inform those who would seek to utilise them.

In Australia, the health system remains largely single-illness oriented despite the prevalence of comorbidity and multi-morbidity [34]. This disease-specific strategy can result in fragmentation of care and will often not address the complex needs of patients with multimorbidity. Initiating systemic changes will require, as a starting point, a strong evidence base that identifies those associated diseases, in order to develop programs of care that cater to such associations and ultimately meet the complex needs of patients with multimorbidity.

### Limitations

A major difference between comorbidity studies is the selected list of diseases, and whether they are self-reported or clinically derived [11]–[16]. Our analysis was based on a set of limited number of self-reported diseases. The prevalence and pattern of comorbidity might have been different had other chronic diseases been included. The accuracy of reported diagnoses is untested, and may be inaccurate for many reasons, including imperfect communication by health professionals, or imperfect memory or understanding by the respondent. There may have been an effect of participants' education levels on correctly understanding and remembering the chronic conditions, which would also influence their answers and potentially the final pattern of the groupings. However, there may also be a relationship between education levels and the diseases actually experienced, and it is not possible to separate the response effects from the clinical effects. While self-reported identification of chronic diseases is criticized by some authors [17] it was found by others to be a well-established method for the measurement of comorbidity and/or multimorbidity [35]. Cluster analysis involves a series of analytic decisions – for instance, about the type of algorithm to be used, measure of similarity (or dissimilarity) to be used, whether clustering is to be done for objects or variables – all these can have an influence on the final results. To address the impact of these decisions we used both object and variable clustering. Moreover, the similarity of groupings between the two clustering procedures and overall similarity of pattern of groups identified using principal component analysis and latent class analysis substantiate the view that our analytical decisions in cluster analysis were appropriate. Although we have endeavored to compare our results with those of similar studies, this comparison is limited by selection of diseases and population age, as outlined above.

### Conclusion

Comorbidity and multimorbidity are an increasingly recognised part of the leading public health problem of managing chronic and complex illness. This area requires more attention and better research. Identification of comorbidity patterns offers valuable information to the stakeholders of health delivery systems and can potentially pave the way to more appropriate health care associated with the pattern and types of multiple diseases. Our results in Table 7 suggests there are three defined groups of chronic diseases: (i) *asthma, bronchitis, arthritis, osteoporosis and depression & anxiety*; (ii) *HBP and diabetes*, and (iii) *cancer*; with *heart disease and stroke* either making a separate group or ‘attaching’ themselves to different groups in different analyses. These findings identified with a range of approaches contribute to a better understanding of the complexity of multimorbidity by characterizing the association between diseases in multiple ways. This study identified some clinically meaningful clusters of multimorbid diseases. However, further studies are needed to provide a strong evidence base on which to formalise groupings which can be more widely used to assist in our understanding of the implications of different comorbidities.
